# Best practices to evaluate the impact of biomedical research software—metric collection beyond citations

**DOI:** 10.1093/bioinformatics/btae469

**Published:** 2024-07-27

**Authors:** Awan Afiaz, Andrey A Ivanov, John Chamberlin, David Hanauer, Candace L Savonen, Mary J Goldman, Martin Morgan, Michael Reich, Alexander Getka, Aaron Holmes, Sarthak Pati, Dan Knight, Paul C Boutros, Spyridon Bakas, J Gregory Caporaso, Guilherme Del Fiol, Harry Hochheiser, Brian Haas, Patrick D Schloss, James A Eddy, Jake Albrecht, Andrey Fedorov, Levi Waldron, Ava M Hoffman, Richard L Bradshaw, Jeffrey T Leek, Carrie Wright

**Affiliations:** Department of Biostatistics, University of Washington, Seattle, WA, 98195, United States; Biostatistics Program, Public Health Sciences Division, Fred Hutchinson Cancer Center, Seattle, WA, 98109, United States; Department of Pharmacology and Chemical Biology, Emory University School of Medicine, Emory University, Atlanta , GA, 30322, United States; Department of Biomedical Informatics, University of Utah, Salt Lake City, UT, 84108, United States; Department of Learning Health Sciences, University of Michigan Medical School, Ann Arbor, MI, 48109, United States; Biostatistics Program, Public Health Sciences Division, Fred Hutchinson Cancer Center, Seattle, WA, 98109, United States; UC Santa Cruz Genomics Institute, University of California Santa Cruz, Santa Cruz, CA, 95060, United States; Roswell Park Comprehensive Cancer Center, Buffalo, NY, 14263, United States; University of California, San Diego, La Jolla, CA, 92093, United States; University of Pennsylvania, Philadelphia, PA, 19104, United States; Jonsson Comprehensive Cancer Center, University of California, Los Angeles, CA, 90095, United States; Institute for Precision Health, University of California, Los Angeles, CA, 90095, United States; Department of Human Genetics, University of California, Los Angeles, CA, 90095, United States; Department of Urology, University of California, Los Angeles, CA, 90095, United States; University of Pennsylvania, Philadelphia, PA, 19104, United States; Division of Computational Pathology, Department of Pathology and Laboratory Medicine, Indiana University School of Medicine, Indianapolis, IN, 46202, United States; Center for Federated Learning, Indiana University School of Medicine, Indianapolis, IN, 46202, United States; Jonsson Comprehensive Cancer Center, University of California, Los Angeles, CA, 90095, United States; Institute for Precision Health, University of California, Los Angeles, CA, 90095, United States; Department of Human Genetics, University of California, Los Angeles, CA, 90095, United States; Department of Urology, University of California, Los Angeles, CA, 90095, United States; Jonsson Comprehensive Cancer Center, University of California, Los Angeles, CA, 90095, United States; Institute for Precision Health, University of California, Los Angeles, CA, 90095, United States; Department of Human Genetics, University of California, Los Angeles, CA, 90095, United States; Department of Urology, University of California, Los Angeles, CA, 90095, United States; University of Pennsylvania, Philadelphia, PA, 19104, United States; Division of Computational Pathology, Department of Pathology and Laboratory Medicine, Indiana University School of Medicine, Indianapolis, IN, 46202, United States; Center for Federated Learning, Indiana University School of Medicine, Indianapolis, IN, 46202, United States; Pathogen and Microbiome Institute, Northern Arizona University, Flagstaff, AZ, 86011, United States; Department of Biomedical Informatics, University of Utah, Salt Lake City, UT, 84108, United States; Department of Biomedical Informatics, University of Pittsburgh, Pittsburgh, PA, 15206, United States; Methods Development Laboratory, Broad Institute, Cambridge, MA, 02141, United States; Department of Microbiology and Immunology, University of Michigan, Ann Arbor, MI, 48109, United States; Sage Bionetworks, Seattle, WA, 98121, United States; Sage Bionetworks, Seattle, WA, 98121, United States; Department of Radiology, Brigham and Women’s Hospital, Harvard Medical School, Boston, MA, 02138, United States; Department of Epidemiology and Biostatistics, City University of New York Graduate School of Public Health and Health Policy, New York, NY, 10027, United States; Biostatistics Program, Public Health Sciences Division, Fred Hutchinson Cancer Center, Seattle, WA, 98109, United States; Department of Biomedical Informatics, University of Utah, Salt Lake City, UT, 84108, United States; Biostatistics Program, Public Health Sciences Division, Fred Hutchinson Cancer Center, Seattle, WA, 98109, United States; Biostatistics Program, Public Health Sciences Division, Fred Hutchinson Cancer Center, Seattle, WA, 98109, United States

## Abstract

**Motivation:**

Software is vital for the advancement of biology and medicine. Impact evaluations of scientific software have primarily emphasized traditional citation metrics of associated papers, despite these metrics inadequately capturing the dynamic picture of impact and despite challenges with improper citation.

**Results:**

To understand how software developers evaluate their tools, we conducted a survey of participants in the Informatics Technology for Cancer Research (ITCR) program funded by the National Cancer Institute (NCI). We found that although developers realize the value of more extensive metric collection, they find a lack of funding and time hindering. We also investigated software among this community for how often infrastructure that supports more nontraditional metrics were implemented and how this impacted rates of papers describing usage of the software. We found that infrastructure such as social media presence, more in-depth documentation, the presence of software health metrics, and clear information on how to contact developers seemed to be associated with increased mention rates. Analysing more diverse metrics can enable developers to better understand user engagement, justify continued funding, identify novel use cases, pinpoint improvement areas, and ultimately amplify their software’s impact. Challenges are associated, including distorted or misleading metrics, as well as ethical and security concerns. More attention to nuances involved in capturing impact across the spectrum of biomedical software is needed. For funders and developers, we outline guidance based on experience from our community. By considering how we evaluate software, we can empower developers to create tools that more effectively accelerate biological and medical research progress.

**Availability and implementation:**

More information about the analysis, as well as access to data and code is available at https://github.com/fhdsl/ITCR_Metrics_manuscript_website.

## 1 Introduction

Biomedical software has become a critical component of biomedical research and enabling major advancements of medicine. Often such software is initially developed so that the developers can use it themselves and then used by others for research (Bitzer *et al.* 2007). However, the life span of biomedical software projects is often cut short because maintenance and continued evaluation is not prioritized by funding institutions or promotion committees (Prlić and Procter 2012). Ultimately the academic infrastructures built around manuscripts are from a time before software and the internet and result in an inefficient ecosystem that rewards new software but does not invest in software that has already been built. This revolving door ultimately undermines the impact that software projects can have on biomedical research and ultimately healthcare. Prioritizing metric collection beyond citations can help funders, promotion committees, and developers to better understand the impact and challenges of software projects (Waller 2018).

To understand current practices and challenges of software developers, we performed a survey of participants in the Informatics Technology for Cancer Research (ITCR) program funded by the National Cancer Institute (NCI). We also manually investigated software among this community to assess how often infrastructure that supports evaluations is implemented and how this impacts rates of papers describing usage of the software. We find that developers recognize the utility of analysing software usage, but struggle to find the time or funding for such analyses. Recognizing the significance of comprehensive software metrics, and providing dedicated funding for developers to robustly collect and analyse such data, would enable biomedical software and the research it supports to achieve drastically greater real-world impact.

### 1.1 Citations alone are not enough

Software with impact is not necessarily highly cited. A study of 4971 academic biomedical and economics articles found that software citations only included version information 28% of the time (Howison and Bullard 2016). Another study evaluating 90 biology articles found that version information was included only 27% of the time and URL information only 17% of the time (Du *et al.* 2021). Specifically, among ITCR-funded software examples, users may forget to cite a tool for visualization, such as the UCSC Xena Genome Browser (Goldman *et al.* 2020). Users might also forget to cite tools used in initial phases of a project, such as EMERSE (Electronic Medical Record Search Engine) (Hanauer *et al.* 2015) which helps identify patient cohorts. Tools which provide access to other software may also not be cited. Examples include Bioconductor (Huber *et al.* 2015), Gene Pattern Notebook (Reich *et al.* 2017), and Galaxy (The Galaxy Community 2022). Understanding system-level tool usage may require looking at individual tools on these platforms. Finally, researchers often only describe a tool without citing it and can do so in unusual locations within manuscripts, such as a figure legend.

Another challenge is that manuscripts for software are a snapshot and do not reflect the evolving nature of the software. Typically, it is much easier to publish manuscripts for new software. However, researchers can save time if they can continue working with tools they are already familiar with.

A new type of manuscript for software updates has been proposed (Merow *et al.* 2023). This could reward developers who start working on software after the initial publication, and provide new ways for funding agencies and others to better recognize software maintenance.

### 1.2 Appropriate use of metrics is the way forward

Metrics beyond citations can be very powerful for continued evaluation and improvements to software. Table 1 explains the benefits of software evaluations for developers, including identifing ways to optimize the tool, to guide future work, to garner funding support, to enhance user commitment, and to motivate community development. Citations alone are insufficient in capturing the dynamic nature of scientific software usage and they are inadequate for helping guide developers to improve their tools.

**Table 1.** Needs, goals, and benefits of software evaluation.

Need	Specific goal	Benefit
Tool optimization	Improving workflows	Identify unexpected usage
		Identify code inefficiencies
		Identify resource usage inefficiencies
		Identify inadequate documentation
		Identify mismatches with defaults and use
	Improve performance	Assess user wait times
		Measure data volume
	Improve usage	Identify software errors
		Identify what features are used and not used
		Identify who the user-base is
		Determine user-base diversity
		Identify sources of other possible users
		Determine what users’ expectations are
		Determine if user expectations are appropriate
		Evaluate success of outreach approaches
	Improve implementation	Identify barriers for adoption
		Identify methods to support adaption
		Identify use of out-dated versions
	Improve usability	Identify user errors
		Identify if and how users are struggling
Tool development & maintenance	Guide future work motivate continued support	Enumerate data types being used
		Discover opportunities for new features
		Discover data needed to address user goals
		Identify more appropriate resource allocation
Gain support	Show evidence of impact	Support future funding requests
		(to maintain or develop new tools)
		Request for resources
		(to maintain or develop new tools)
Gain user commitment	Evidence of tool acceptance	Reassure users about tool to:
		– Promote continued use
		– Promote usage of new tools by the same developers
		– Promote usage by more diverse users
Gain community development	Evidence of co-development	Encourage contributions

Software evaluation can support identification for tool optimization and development and can demonstrate tool value to others.

Evaluation metrics for the purpose of continued development can include the number of new users, returning users, and total downloads of the software, but the types of possible metrics vary based on the type of tool and context (see Table 2). These and other metrics can allow assessment of the rate of establishment within a community. Proper metrics should not only examine the software’s performance but assess if motivations and goals of the users using the software are being met. Ideally metrics also help gather information about the downstream impact of the tool on biomedical research.

**Table 2.** Example metrics.

Measure	Example metrics	Use
Tool dissemination	• Total unique downloads (Thelwall and Kousha 2016, Eisty *et al.* 2018, Zhao *et al.* 2021) • New users (Begany *et al.* 2021, Sayyed-Alikhani *et al.* 2021) • Returning users (Begany *et al.* 2021, Sayyed-Alikhani *et al.* 2021) • Download count by version (Rossi *et al.* 2010, Howison *et al.* 2015)	• Determining popularity of a given tool
	• Download count by version	• Assessing if users are keeping up-to-date
Tool usefulness	• Number of software engagements by user (Begany *et al.* 2021, Chang *et al.* 2021)	• Determining prevalence of usage
Tool reliability	• Proportion of runs without a crash or error (Eisty *et al.* 2018, Hunter-Zinck *et al.* 2021) • Test coverage (Hunter-Zinck *et al.* 2021)	• Improving error handling, bug fixes
Tool versatility	• Distribution of data types (inferred from metadata) (Eisty *et al.* 2018)	• Improving tool flexibility & generalizability
Interface acceptability	• Proportion of visitors who actually engage with the tool (Kumar and Hasteer 2017, Ramakrishnan and Gunter 2017) • User error frequency (Kumar and Hasteer 2017, Eisty *et al.* 2018)	• Graphical tool and website acceptability
Performance	• Maximum memory usage (Eisty *et al.* 2018) • Average time–to–complete of algorithmic steps (Eisty *et al.* 2018)	• Requirements analysis • Tuning

A variety of metrics can be used to attempt to interpret to usefulness, reliability, and uptake by the community and more. Here, we describe metrics used by the authors of the paper. See Lenarduzzi *et al.* (2020), Eisty *et al.* (2018), Thelwall and Kousha (2016) for more information about metrics used by others.

Despite these strengths, developers and funders must understand challenges and nuance in interpreting these metrics. Communities like CHAOSS (Community Health Analytics in Open Source Software) have focused on the proper collection, evaluation, and standards for software metric collection https://chaoss.community/. In an effort to have more expansive metrics adopted by the biomedical research community, we aim to provide guidance for evaluations of software impact and engagement. We also discuss ethical considerations and challenges of such evaluations that still require solutions. The guidance presented here holds the potential for developers to improve the use and utility of their tools, improve their chances of funding for future development, and ultimately lead to the development of even more useful software to advance science and medicine (Wratten *et al.* 2021).

## 2 Materials and methods

We performed two analyses to get a sense of software evaluation within the community of developers of the ITCR program funded by the NCI. Our first surveyed developers to better understand how they think about software evaluation. Our second aimed to determine what infrastructure is often implemented to support software evaluation and if such implementation was associated with the frequency of papers describing usage of the software (see Supplemental Note S1).

In the first analysis, we surveyed 48 ITCR participants. Limited time (68% of respondents) and funding (57% respondents) were major barriers for performing software impact evaluations (respondents could select multiple barriers). Although a few funding mechanisms support the maintenance and analysis of software (as opposed to creation of new software), such as the ITCR sustainment awards (Kibbe *et al.* 2017, Warner and Klemm 2020), or the Essential Open Source Software for Science program of the Chan Zuckerberg Initiative (Science 2019), more funding for software sustainability is needed compared to what is currently available. Awareness of this need was also demonstrated by the recent Declaration on Funding Research Software Sustainability by the Research Software Alliance (ReSA) (Barker *et al.* 2023). While scientific software has become critical to most researchers, the funding to support the maintenance of such software is not reflective of the current level of usage (Siepel 2019). The next major barriers were privacy concerns (38% of respondents), technical issues (32% of respondents), and not knowing what methods to use for evaluations (27% of respondents). Despite these apparent challenges, 73% of respondents state that such evaluations have informed new development ideas, 60% stated that it informed documentation, and 54% stated that it helped justify funding (respondents could select multiple benefits). Thus, additional support for evaluations of software usage and impact could greatly benefit the continued development of software. Responses to an open-ended question asking “Is there anything you would like to measure but have been unable to capture?” included (each of these examples were unique responses): collaborations that the tool supported, the number of commercial applications using the tool, the fraction of assumed user base that actually uses the tool, the downstream activity—what do users do with the results, and user frustration. These responses outline many of the challenges that developers often face. See Supplemental Table S1 and Supplemental Note S2 for examples of the goals of the respondents.

We also manually inspected 44 scientific research tools, 33 of which were funded by ITCR alone, and seven funded by the Cancer Target Discovery and Development (CTD^2^) Network (Aksoy *et al.* 2017), as well as four tools funded by both. Each were inspected for infrastructure that could help users know about the tool or how to use it, as well as possible infrastructure related to software health metrics that indicate how recently the code was rebuilt or tested (Srivastava and Schumann 2011). We then investigated if there were any associations with these aspects and usage. A variety of different types of research-related tools or resources were inspected—see Table 3. Each tool or resource was manually inspected (by someone not involved in developing these tools) to get the experience of a potential user briefly examining related websites to determine if the tool had: a DOI for the software itself, information on how to cite the software, information on how to contact the developers, documentation (and how much), an X/Twitter presence, and badges about software health metrics (such as those related to maintenance and testing) (Srivastava and Schumann 2011) visible on a related website.

**Table 3.** Scientific tools and resources evaluated.

Type	Description	Count	Percentage
Plug-in/extension	These tools are plug-in or extension software that adds functionality to other software	2	4.5
Jupyter/Python	These tools are scripts written in Python or Jypyter Notebooks	5	11.4
Database/Ontology	These tools provide users access to data or standards	5	11.4
Computing platforms	These tools allow users to upload data and perform analysis on a cloud or server	5	11.4
Web-based tools	These tools are hosted on a website where users can access the tool and use it	6	13.6
Desktop applications/command-line tools	These tools require users to download the tool to their computer or server, desktop applications may or may not require command-line interactions, while command-line tools do	9	20.5
R packages	Software written in the R programming language	12	27.3
Total	NA	44	100

Here, we show the variety among the 44 ITCR and CTD^2^ scientific research tools/resources evaluated for various characteristics by manual inspection for infrastructure used to support software evaluation metrics beyond software paper citations.

**Figure 1.**
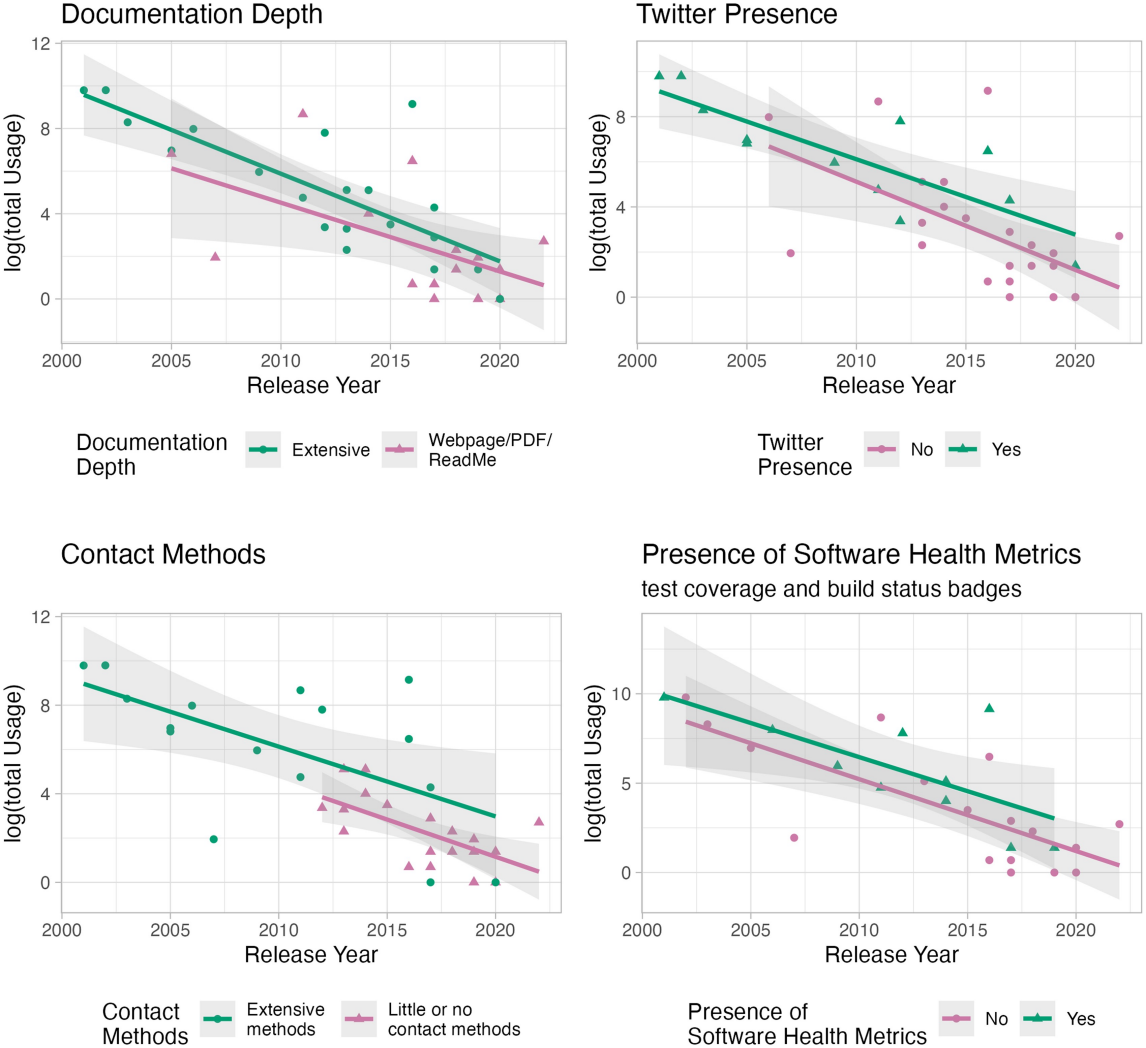
Aspects of software infrastructure appear to be associated with a larger number of published manuscripts from users describing usage of the software in the SoftwareKG-PMC database. The *X*-axis indicates the age of the software by showing the year that it was released. The *Y*-axis indicates the log of the total number of papers that describe usage of the software in the SoftwareKG-PMC database. See Supplementary material and our website for more information.

To evaluate a proxy for usage, we used the SoftwareKG-PMC database (Krüger and Schindler 2020), which does not include citations to tools, only plain-text mentions inferred by a text-mining algorithm. This was to enable us to capture cases where users may have mentioned but not necessarily cited a tool. Importantly, mentions also do not always indicate usage. The database does not know anything about these tools per se, and not all of these mentions necessarily correspond to the same tool. For example, DANA is an ITCR tool for microRNA analysis but there are other tools with the same name. Although time since the tool release was the largest contributor to variation in the number of papers describing usage, various aspects of infrastructure that could help users know about a tool (social media on twitter), have confidence in the tool (badges about software builds or tests), or learn more about how to use the tool (extensive documentation and feedback mechanisms) all seemed to be associated with an increased rate of manuscripts that described using the tool. All show significant association (*P* < 0.05) with usage when not accounting for tool age. Only having extensive feedback mechanisms was significantly associated when also accounting for tool age (see Fig. 1). For more information about this analysis, see our website https://hutchdatascience.org/ITCR_Metrics_manuscript_website/.

## 3 Results

The results of our evaluation of scientific software suggests that infrastructure can support the collection of more metrics and support more mentions of software in papers. Specifically, our results showed that active social media, more in-depth documentation, clear methods to contact developers, and software health metrics [metrics related to how often the software is tested, developed, etc (Srivastava and Schumann 2011)] appear to enhance mentions in papers.

The infrastructure described in Table 4 and Supplemental Note S3 could enable more comprehensive metrics about insights regarding software usage and impact. Funders and developers should consider these elements when considering the impact and new directions for a project.

**Table 4.** Software infrastructure can enable the capture of valuable metrics for evaluating engagement and impact.

Elements	Options	Tools to enable metric collection	Possible enabled metrics	Considerations
Web presence	Web-based tool	• Cronitor for tools using cron job scheduling (Peters 2009)) • Google Analytics	• Identify where your tool is being used • Possibly identify what data are being used	May need to consider privacy restrictions for tracking IP addresses
	Documentation website	• Google Analytics	• Counts of page views and scrolls	Pages with more views may identify widely used features or confusing aspects
Citability	Provide a way to cite such as a direct software DOI (Fenner 2018), as well as publishing software manuscripts and information on how to cite, can help people to cite your software	• To create DOIs: Zenodo, Dryad, Synapse, and Figshare • To track DOIs: Altmetric	• Total citation counts • Counts of citations by journals of different fields	Semantic Scholar provides reports that indicate where citations have occurred within scientific articles. Direct DOIs for software (in addition to software manuscripts) is a useful and recognized method for allowing others to cite software, especially when manuscripts are not yet published. See Smith *et al.* (2016) for best practices. Not all DOI managers are created equally and some have more verification processes (Amorim *et al.* 2015)
Contact	Feedback mechanisms	• GitHub issue templates • Surveys	• User feedback count • Addressed user feedback count	Often users will only provide feedback if something is broken. Depending on the tool, many users may not be comfortable with GitHub Issues
	Discussion forums	• Discourse • Biostar (Parnell *et al.* 2011) • Bioinformatics Stack Exchange • Google Groups	• Metrics based on user engagements and answered questions	Forums can illustrate the amount of community activity with a particular tool (Parnell *et al.* 2011, Howison *et al.* 2015). They can also save time for development as users help each other instead of developers answering individual emails for repeat problems (Prlić and Procter 2012). A code of conduct can help create a supportive community
	Newsletter emails	• Mailchimp • HubSpot	• Count of newsletter openings • Count of link clicks • Count of unsubscribers	Newsletters can help inform users about new features
Usability testing	• Observe a few people use the tool • Discussion forums	• Zoom screen sharing and recording • Discussion Forums (above)	• Qualitative information about how users interact with your software	Even low numbers of usability interviews (3) can yield fruitful lessons that can be paired with other metrics to guide development. Forums that provide Q&A can identify usability issues and bugs (Howison *et al.* 2015, Patrick 2020)
Workshops	• Online or in-person • Basics or new features	• Attendees can participate in surveys	• Quantity, duration, and attendance at workshops are metrics that can be reported to funding agencies	Recordings can be posted on social media (for additional metrics).
Social media	• YouTube videos • Twitter/Mastodon • Instagram • LinkedIn	• Hootsuite—social media management	• Engagement metrics (video watch counts, likes, etc.)	Pairing social media metrics with software engagement metrics can determine if outreach strategies are successful
Reviews	Review forum	• SourceForge • GitHub	• Stars • Watchers • Forks • Number of reviews	Positive reviews, active community participation, and code review can be reassuring to funders and users alike

Note that there are other helpful tools to enable metric collection. These are simply examples based on the experience of software developers funded by ITCR, e.g. the developers of QIIME 2 (Bolyen *et al.* 2019) found metrics from workshops, forums, and other forms of outreach valuable for evaluating community uptake and user experience. Altmetric at https://zenodo.org/, Google Analytics at https://docs.github.com/en/actions, Bioinformatics Stack Exchange at ttps://bioinformatics.stackexchange.com, Cronitor at https://cronitor.io, Discourse at https://www.discourse.org/, Dryad at https://datadryad.org/, Figshare at https://figshare.com, Google Groups at https://support.google.com/groups, Hootsuite at https://www.hootsuite.com/, HubSpot at https://www.hubspot.com, Mailchimp at https://mailchimp.com/, Semantic Scholar at https://www.semanticscholar.org/, Singularity at https://sylabs.io/, Sourceforge at https://sourceforge.net/, Synapse at https://www.synapse.org/, and Zenodo at https://zenodo.org/.

## 4 Discussion

With new metrics collected through the software infrastructure described in Table 4 comes a new host of challenges that require guidance. Here, we layout how the metrics collected from the infrastructure discussed in the previous section should be handled appropriately. The following are guidance based on the composite experience of the authors:

### 4.1 Successful evaluations are anchored by an understanding of the intended use of the software

The intended goal or purpose of the scientific software should inform how the software is evaluated (Basili *et al.* 1994). Computational tools are designed to support well-defined goals often called use cases (Gamma *et al.* 1995) for specific sets of users called personas (Cooper 2004). Efforts to evaluate the impact of tools should be guided by a clear understanding of these, use cases and personas to assess how well the tools meet the intended goals and for all intended users.

### 4.2 Metric selection should be hypothesis driven

Collecting an exhaustive amount of user data before selecting metrics can increase the risk that metrics are selected in a biased manner. This can lead to picking metrics that look good but are not necessarily as meaningful to the intended usage of the tool. To mitigate this, metrics can be selected ahead of time based on a specific hypothesis to ultimately evaluate how well the software supports its intended goals (Mullen 2020).

### 4.3 No single evaluation method works for every type of software

No individual scheme for collecting metrics fits every type of software tool. The meaning of a set of metrics may differ across contexts. For example, the location of a tool (e.g. on the web or downloaded) can influence user access to software versions and how one might collect metrics. For a web-based application, users will rarely have access to older versions. Thus, developers can add version updates and collect metrics with clarity about how usage changed. For locally run tools, users may be using older, previously-downloaded versions. Additionally, tools that are installed on institutional servers have much smaller installation counts than those installed on individual computers. No one metric is one size fits all and each software tool must be thoughtfully planned out for how it should be evaluated.

### 4.4 Metrics require interpretation

Metric interpretation is rarely straightforward. A spike may correspond to a workshop using the tool or a recent publication citing the tool. Negative trends may indicate a break in the academic calendar, holidays, down time of a host server, or software bugs. It is also important to avoid comparisons between metrics for tools with different users and contexts.

Total unique downloads might indicate software popularity, but does not tell us whether users found it useful. Instead, metrics about returned usage by the same users or the number of launches of the software over a certain predefined session time threshold may better evaluate actual usage. For tools that offer access or analyses of different data types, one may want to parse usage by data types to evaluate how useful the tool appears to support different kinds of users. Specific measures can provide a common basis comparing versions and potentially against other similar software.

### 4.5 Metrics of best practices provide indicators of software health

Tracking adherence to best practices of software engineering can be a useful way to assess software project health Srivastava and Schumann (2011), including the use of version control systems, high coverage of code with testing, and use of automated or continuous integration. None of these measures of project health are perfect (and can be done poorly) but can collectively indicate software health. Including badges for such indicators on code repositories and websites can give users and others confidence. Some software packages can help automatically assess package health like the riskmetric package (https://pharmar.github.io/riskmetric/) for evaluation of R packages (R Validation Hub *et al.* 2024). Additional detail on these topics, can be found in The Pragmatic Programmer(Thomas and Hunt 2019). See Table 5 and Supplemental Note S4 for suggestions.

**Table 5.** Software health infrastructure.

Infrastructure	Options	Tools to enable metric collection	Possible enabled metrics	Significance for users, developers, and funders
Version control	Without automation	• Git/GitHub (The insight tab and API allow for systematic metric collection) • Git/GitLab • BitBucket	• Commit frequency (how often code is updated) • Date of the most recent commit • Number of active contributors • Software versions updates	Commit frequency allows assessment of how actively the software is being maintained. The number of contributors can indicate sustainability. One single contributor may pose a sustainability risk. Version information can enable users to determine if they are using the most up-to-date version. Developers can utilize these metrics to determine which projects need more attention and to garner support from funding agencies to prove that they have done a thorough job developing and maintaining the software
	With automations	• GitHub actions • Travis CI • CircleCI	• Current build status (if the software built without errors)	Continuous integration and continuous deployment or delivery are terms to describe a situation where every time code is modified, the full code product is automatically rebuilt or compiled. Continuous deployment or delivery describes the automatic release of this new code to users. Delivery in this case describes situations where the software requires more manual releases while deployment is seamless. GitHub Actions can also help with metric collection from the GitHub API. Developers can use build status to understand if the software is performing appropriately. This can help funders to recognize how well a tool is working and being developed and maintained
Testing	Automated testing	• GitHub Actions • Travis CI • CircleCI	• Test code coverage (the fraction of lines of code in the project that are covered by tests)	Unit tests check individual pieces of code; component and integration tests check that pieces of code behave correctly together; acceptance tests check the overall software behaviour. Achieving in-depth test coverage requires careful software design. Test coverage does not evaluate the quality of the test cases or assertions. Test metrics can help users, developers, and funders understand how thoroughly and robustly the code has been assessed for aberrant behaviour
Licensing	A variety of licenses exist to allow or disallow reuse and to require attribution	• Creative commons	• Possible quantification of reuse of your software code	Clearly indicating if and how people can reuse your code will make them more comfortable to do so. Determining when this is done can be a challenge, but requiring attribution makes this more feasible. This can indicate to users and funders if the developers have carefully considered the downstream use of their code outside of their own software. This can better enable additional tools to be built using your code and can help you to track usage if you use a license that requires attribution for reused coded

Infrastructure that enables collecting metrics about software health (meaning how robustly software was built and maintained) (Srivastava and Schumann 2011), can reassure users and funders. Bitbucket at https://bitbucket.org/product, CircleCI at https://circleci.com/docs, Creative Commons at https://creativecommons.org/licenses/, GitLab at https://about.gitlab.com/, GitHub at https://github.org, GitHub actions at https://docs.github.com/en/actions, and Travis CI at https://config.travis-ci.com/.

### 4.6 Metrics related to software quality and reusability could reassure users and funders

Software reusability metrics have been suggested to enable better discernment of the capacity for code to be reused in other contexts. These metrics can also evaluate if code is written to be more resilient over time to dependency changes and other maintenance challenges. One example would be the degree to which aspects of the software are independent of one another (Mehboob *et al.* 2021). As research funders start to value software maintenance more, metrics related to resilience and reusability may become more valuable. Other similar metrics related to maintainability have been used in the software community for some time relying on metrics such as the number of code comments, lines of code, or code complexity metrics (Wang 2006), but open source software projects with community contributors can make aspects related software maintainability a challenge (Oman and Hagemeister 1994, Welker 2001, Ganpati *et al.* 2012).

## 5 Challenges and nuances

Here, we outline a number of challenges and nuances associated with evaluating metrics for software usage and impact.

### 5.1 Distorted metrics

Projects like the ITCR-funded Bioconductor (Huber *et al.* 2015), with a large variety of software packages, offer an opportunity to assess distortion of metrics by evaluating how different packages are used over time, revealing important nuances (see Table 6). Overall major themes seen include, accidental usage by scripts that accidentally loop through downloading a piece of software many times, usage of software to support other software for technical reasons, as well as unexpected patterns of persistent use after a tool is theoretically no longer as useful. This is believed to be due to downloads on servers using lists of historically typically used packages. Finally, background levels of usage with low levels of downloads even for tools that are no longer supported.

**Table 6.** Distorted metrics.

Distortion	Example
Accidental usage	Occasionally scripts used on servers may inadvertently download a package repeatedly and rapidly hundreds to thousands of times, resulting in distorted download metrics that are not representative of real usage. Unique IP download information is useful to distinguish between one user downloading many times versus many users a few times. Given privacy concerns, an alternative solution could involve tracking the timing and approximate location of downloads with a threshold for what would be more than expected as maximum real usage, like a group of people following a tutorial
Background usage	There is a baseline background level of downloads across all packages in Bioconductor (including those that are no longer supported). Thus, if a new package has 250 downloads in the first year this may seem like a successful number, but actually it is similar to background levels
Technical versus research usage	It can be difficult to discern if the usage of a package is for scientific research itself or supporting the implementation of other software. While both are arguably valuable, distinguishing between these motivations can help us understand a particular software’s impact in a field. For example, the S4Vectors package (10.18129/B9.bioc.S4Vectors) (Pagès *et al.* 2022) is an infrastructure package used by many other packages for technical and non-biological reasons and is therefore not often directly downloaded by end-users. This package is also included in automated checks for other Bioconductor packages using GitHub actions. Another example of support implementation is in the context of container image use. Containerization software [like Docker (https://www.docker.com/) and Singularity] often install software packages for individual environments that could inflate usage metrics statistics. For instance, a user who is actively developing a container may re-trigger the build and thus installation of associated software many times over the course of a project
Usage persistence	The affy package (10.18129/B9.bioc.affy) (Gautier *et al.* 2004) was one of the early packages for microarray analysis, a technology that has largely been replaced by newer technologies, which can be seen by the rate of microarray submissions to GEO overtime. However, despite the field transitioning away from microarray methods (Mantione *et al.* 2014), the package was downloaded in 2021 at rates that doubled the rates in 2011. The authors speculate that this could be due to people historically requesting that affy be installed on servers and that this is just persisting, or perhaps it is being used for preliminary hypothesis testing using existing micrarray data, or perhaps it is being used because other microarray packages are no longer supported

Here, we provide more in-depth information about metric distortion themes identified evaluating tools in Bioconductor (which is ITCR-funded). GEO = Gene Expression Omnibus.

### 5.2 Clinical data challenges

Clinical data often contain protected health information (PHI). Thus, the number of individuals that have access to the data is generally smaller. Many tools containing clinical data are also run at an enterprise level (such as the ITCR-funded tool, EMERSE), meaning they are installed only one time by system administrators and accounts are provisioned to users. Thus, counting installations does not represent the overall use. Further, security mechanisms to protect clinical data inhibit developers from accessing the installed systems themselves. Ultimately, due to downloads typically being at an institutional level for clinical tools, metrics around software downloads underestimate their impact. It would not be realistic to compare the usage metrics of such tools to more widely available and accessible tools.

### 5.3 Goodhart’s law

Goodhart’s law states that “every measure which becomes a target becomes a bad measure” (Hoskin 1996). For example, h-indices (the number of papers an author has with that many or more citations) are used to assess an author’s impact. As the h-index grew in popularity, the number of researchers included as coauthors, the number of citations per paper, and the fraction of self-citations increased, each leading to an increased h-index. Although metrics could be used to bring about best practices for binary outcomes (i.e. public deposition of code), for more quantitative metrics (e.g. number of downloads) the results could easily become meaningless. The impact behind this concept cannot be entirely avoided because of fundamentals of human behaviour but one way to minimize this effect is to continue to evaluate metrics over time, to consider if our metrics are truly measuring what we think they are, to consider if our metrics are actually fair to a diverse range of projects, and to consider new metrics as needed (Fire and Guestrin 2019). Funding agencies need to consider how each type of tool is context-dependent, and that impact should be compared between similar classes of tools.

### 5.4 Security, legal and ethical considerations

Often with phone-home software (the collection of information from the computers of users that downloaded or installed a particular software) or web-based analytics, users are tracked for specific usage. Occasionally software developers will notify users that they are being tracked, however this is often not required. The General Data Protection Regulation (GDPR), implemented in 2018, requires that organizations anywhere in the world respect certain data collection obligations regarding people in the European Union. It is intended to protect the data privacy of individuals and mostly guards against the collection of identifiable personal information. Data collection of software usage needs to be mindful of the GDPR and any other international regulations. As science is particularly an international pursuit, often users may reside outside the country where the tool was developed.

One way to mitigate this is to let users choose if they wish to be tracked. Developers can also design tracking to be more anonymous. A genome visualization tool may track the number of unique uses, but not track what part of the genome was visualized [as is the case for the UCSC Xena Genome Browser (Goldman *et al.* 2020)]. Google Analytics (https://marketingplatform.google.com/about/analytics/) provides support to mask unique IP addresses of visitors to a website tracked by the system. Ethical and legal consequences should be considered when designing or implementing tracking systems of software (see Supplemental Note S5 for more information).

### 5.5 Conclusions

Our assessments indicate that cancer software developers of the ITCR find it difficult to find the time or funding to evaluate the impact and usage of their software using metrics, despite their awareness of the benefits. Many have found such evaluations useful for driving future development and obtaining additional funding. A sizable portion (27%) of those surveyed self-reported as not knowing what methods to use for such evaluations. We also find from our manual evaluation of a subset of scientific software tools that tools appear to be more widely used when developers provide deeper documentation, badges about software health metrics, and more in-depth contact information, as well as having a Twitter presence. It is not clear why this is. It may be that those who have the time and support to more thoroughly document and advertise their tools may also have more resources to developer the tool itself, lending to wider usage. However, it may also be that a social media presence brings new users to tools and that the other infrastructure (badges, deeper documentation, etc.) help new users to trust software. Further studies are necessary to understand these patterns. However, it suggests that supporting developers to spend more time on such elements could drive further usage of existing tools. We hope that funding agencies will value supporting developers to evaluate, promote, and maintain existing tools in addition to the current typical model for most agencies to prioritize the creation of new tools. A recent article (Merow *et al.* 2023) suggested that a new type of manuscript for software updates may help the field to better reward maintenance of existing software. We argue that inclusion of evaluations of software impact and usage could also be incorporated into such a model for software-related manuscripts.

While metric collection beyond traditional citations is only one piece of the software development workflow, we feel that it has been underappreciated by funding institutions and promotion committees. In addition, while common metrics may be valuable for comparisons of similar types of tools, other types of metrics may give more insight about the downstream impact of a tool in terms of what development and advancements in the field that the software supported. For example, perhaps we should consider how much a software tool inspires the development of other tools, the value of the papers that cite a tool (perhaps by citation rate, measures of innovation, or measures of clinical impact, such as clinical trials) Certainly as scientific software continues to be critical for scientific and medical advancement, we should continue to think beyond the software citation model and consider the infrastructure and metrics we have discussed here as we determine how to support scientific software developers in the future.

## Supplementary Material

btae469_Supplementary_Data

## Data Availability

More information about the analysis, as well as access to all data and code is available at https://hutchdatascience.org/ITCR_Metrics_manuscript_website/ and https://github.com/fhdsl/ITCR_Metrics_manuscript_website.

## References

[btae469-B1] Aksoy BA , Dančík V, Smith K et al CTD2 dashboard: a searchable web interface to connect validated results from the Cancer Target Discovery and Development Network. Database (Oxford) 2017;2017:bax054. 10.1093/database/bax054 29220450 PMC5569694

[btae469-B2] Amorim RC , Aguiar Castro J, Silva JRD et al A comparative study of platforms for research data management: interoperability, metadata capabilities and integration potential. In: R Alvaro, C Ana Maria, C Sandra, R Luis Paulo (eds.), New Contributions in Information Systems and Technologies, Advances in Intelligent Systems and Computing, Vol. 353. Cham: Springer International Publishing, 2015, 101–111. 10.1007/978-3-319-16486-1_10

[btae469-B3] Barker M , Chue Hong NP, van Eijnatten J et al Amsterdam Declaration on Funding Research Software Sustainability. Zenodo, 2023. 10.5281/zenodo.7740084

[btae469-B4] Basili VR , Caldiera G, Rombach DH. The Goal Question Metric Approach, Volume I. United Kingdom: John Wiley & Sons, 1994.

[btae469-B5] Begany GM , Martin EG, Yuan XJ. Open government data portals: predictors of site engagement among early users of health data NY. Gov Inform Quart 2021;38:101614.

[btae469-B6] Bitzer J , Schrettl W, Schröder PJH. Intrinsic motivation in open source software development. J Comp Econ 2007;35:160–9. 10.1016/j.jce.2006.10.001

[btae469-B7] Bolyen E , Rideout JR, Dillon MR et al Reproducible, interactive, scalable and extensible microbiome data science using QIIME 2. Nat Biotechnol 2019;37:852–7. 10.1038/s41587-019-0209-9 31341288 PMC7015180

[btae469-B8] Chang H-Y , Colby SM, Du X et al A practical guide to metabolomics software development. Anal Chem 2021;93:1912–23. 33467846 10.1021/acs.analchem.0c03581 PMC7859930

[btae469-B9] Cooper A. Inmates Are Running the Asylum, The: Why High-Tech Products Drive Us Crazy and How to Restore the Sanity. 2nd edn. Carmel, Indiana, United States: Sams, 2004.

[btae469-B10] Du C , Cohoon J, Lopez P et al Softcite dataset: a dataset of software mentions in biomedical and economic research publications. J Assoc Inf Sci Technol 2021;72:870–84. 10.1002/asi.24454

[btae469-B11] Eisty NU , Thiruvathukal GK, Carver JC. A survey of software metric use in research software development. In: *2018 IEEE 14th International Conference on e-Science (e-Science)*, Amsterdam, the Netherlands. Piscataway, NJ, United States: IEEE, 2018, 212–222. 10.1109/eScience.2018.00036

[btae469-B43] Essential Open Source Software for Science. Chan Zuckerberg Initiative, November 2019. https://chanzuckerberg.com/rfa/essential-open-source-software-for-science/.

[btae469-B12] Fenner M. *DOI Registrations for Software*. 2018. https://datacite.org/blog/doi-registrations-software/

[btae469-B13] Fire M , Guestrin C. Over-optimization of academic publishing metrics: observing Goodhart’s Law in action. GigaScience 2019;8:giz053. 10.1093/gigascience/giz053 31144712 PMC6541803

[btae469-B14] Gamma E , Helm R, Johnson R et al Design Patterns: Elements of Reusable Object-Oriented Software. Boston, MA, United States: Addison-Wesley, 1995.

[btae469-B15] Ganpati A , Kalia A, Singh H. A comparative study of maintainability index of open source software. Int J Emerg Technol Adv Eng 2012;2(10):228–30.

[btae469-B16] Goldman MJ , Craft B, Hastie M et al Visualizing and interpreting cancer genomics data via the Xena platform. Nat Biotechnol 2020;38:675–8. 10.1038/s41587-020-0546-8 32444850 PMC7386072

[btae469-B17] Hanauer DA , Mei Q, Law J et al Supporting information retrieval from electronic health records: a report of University of Michigan’s nine-year experience in developing and using the Electronic Medical Record Search Engine (EMERSE). J Biomed Inform 2015;55:290–300. 10.1016/j.jbi.2015.05.003 25979153 PMC4527540

[btae469-B18] Hoskin K. The “awful idea of accountability”: inscribing people into the measurement of objects. In: Munro R, Mouritsen J (eds.), Accountability: Power, Ethos and the Technologies of Managing. London: International Thomson Business Press, 1996.

[btae469-B19] Howison J , Bullard J. Software in the scientific literature: problems with seeing, finding, and using software mentioned in the biology literature. J Assoc Inf Sci Technol 2016;67:2137–55. 10.1002/asi.23538

[btae469-B20] Howison J , Deelman E, McLennan MJ et al Understanding the scientific software ecosystem and its impact: current and future measures. Res Eval 2015;24:454–70. 10.1093/reseval/rvv014

[btae469-B21] Huber W , Carey VJ, Gentleman R et al Orchestrating high-throughput genomic analysis with Bioconductor. Nat Methods 2015;12:115–21. 25633503 10.1038/nmeth.3252 PMC4509590

[btae469-B22] Hunter-Zinck H , de Siqueira AF, Vásquez VN et al Ten simple rules on writing clean and reliable open-source scientific software. PLoS Comput Biol 2021;17:e1009481. 10.1371/journal.pcbi.1009481 34762641 PMC8584773

[btae469-B23] Kibbe W , Klemm J, Quackenbush J. Cancer informatics: new tools for a data-driven age in cancer research. Cancer Res 2017;77:e1–2. 10.1158/0008-5472.CAN-17-2212 29092926

[btae469-B24] Krüger F , Schindler D. A literature review on methods for the extraction of usage statements of software and data. Comput Sci Eng 2020;22:26–38. 10.1109/MCSE.2019.2943847

[btae469-B25] Kumar R , Hasteer N. Evaluating usability of a web application: a comparative analysis of open-source tools. In: *2017 2nd International Conference on Communication and Electronics Systems (ICCES).* 2017, 350–4. 10.1109/CESYS.2017.8321296

[btae469-B26] Gautier L , Cope L, Bolstad BM et al affy—analysis of *Affymetrix GeneChip* data at the probe level. Bioinformatics 2004;20:307–15. 10.1093/bioinformatics/btg405 14960456

[btae469-B27] Lenarduzzi V , Taibi D, Tosi D et al Open source software evaluation, selection, and adoption: a systematic literature review. In: *2020 46th Euromicro Conference on Software Engineering and Advanced Applications (SEAA)*, Portoroz, Sloveni, August 2020, 437–44, Piscataway, NJ, United States: IEEE. 10.1109/SEAA51224.2020.00076

[btae469-B28] Mantione KJ , Kream RM, Kuzelova H et al Comparing bioinformatic gene expression profiling methods: microarray and RNA-Seq. Med Sci Monit Basic Res 2014;20:138–42. 10.12659/MSMBR.892101 25149683 PMC4152252

[btae469-B29] Mehboob B , Chong CY, Peck Lee S et al Reusability affecting factors and software metrics for reusability: a systematic literature review. Softw Pract Exp 2021;51:1416–58. 10.1002/spe.2961

[btae469-B30] Merow C , Boyle B, Enquist BJ et al Better incentives are needed to reward academic software development. Nat Ecol Evol 2023;7:626–7. 10.1038/s41559-023-02008-w 36849538

[btae469-B31] Mullen C. Hypothesis-Driven Development. MIT Lincoln Laboratory 2020, https://www.ll.mit.edu/sites/default/files/project/doc/2020-07/Hypothesis-Driven%20Development_v4.pdf.

[btae469-B32] Oman P , Hagemeister J. Construction and testing of polynomials predicting software maintainability. J Syst Softw 1994;24:251–66. 10.1016/0164-1212(94)90067-1

[btae469-B33] Pagès H , Lawrence M, Aboyoun P. S4Vectors: foundation of vector-like and list-like containers in Bioconductor. R package version 0.34.0. 2022, https://bioconductor.org/packages/release/bioc/html/S4Vectors.html.

[btae469-B34] Parnell LD , Lindenbaum P, Shameer K et al BioStar: an online question & answer resource for the bioinformatics community. PLoS Comput Biol 2011;7:e1002216. 10.1371/journal.pcbi1002216 22046109 PMC3203049

[btae469-B35] Patrick MT. Exploring software reusability metrics with Q&A forum data. J Syst Softw 2020;168:110652.

[btae469-B36] Peters R. cron. In: P Ron (ed.), Expert Shell Scripting. Berkeley, CA: Apress, 2009, 81–85. 10.1007/978-1-4302-1842-5_12

[btae469-B37] Prlić A , Procter JB. Ten simple rules for the open development of scientific software. PLoS Comput Biol 2012;8:e1002802. 10.1371/journal.pcbi.1002802 23236269 PMC3516539

[btae469-B38] R Validation Hub Kelkhoff D , Gotti M et al riskmetric: Risk Metrics to Evaluating R Packages. R package version 0.2.4.9000. 2024, https://cran.r-project.org/web/packages/riskmetric/index.html.

[btae469-B39] Ramakrishnan L , Gunter D. Ten principles for creating usable software for science. In: *2017 IEEE 13th International Conference on e-Science (e-Science)*. 2017, 210–8. 10.1109/eScience.2017.34

[btae469-B40] Reich M , Tabor T, Liefeld T et al The GenePattern notebook environment. Cell Syst 2017;5:149–51.e1. 10.1016/j.cels.2017.07.003 28822753 PMC5572818

[btae469-B41] Rossi B , Russo B, Succi G. Download patterns and releases in open source software projects: a perfect symbiosis? In: *Open Source Software: New Horizons: 6th International IFIP WG 2.13 Conference on Open Source Systems, OSS 2010, Notre Dame, IN, USA*, *May 30–June 2, 2010. Proceedings 6*. Springer, 2010, 252–67.

[btae469-B42] Sayyed-Alikhani A , Chica M, Mohammadi A. An agent-based system for modeling users’ acquisition and retention in startup apps. Exp Syst Appl 2021;176:114861.

[btae469-B44] Siepel A. Challenges in funding and developing genomic software: roots and remedies. Genome Biol 2019;20:147. 10.1186/s13059-019-1763-7 31358028 PMC6664559

[btae469-B45] Smith AM , Katz DS, Niemeyer KE; FORCE11 Software Citation Working Group. Software citation principles. PeerJ Comput Sci 2016;2:e86. 10.7717/peerj-cs.86

[btae469-B46] Srivastava AN , Schumann J. The case for software health management. In: *2011 IEEE Fourth International Conference on Space Mission Challenges for Information Technology*. Palo Alto, CA, United States, August 2011, 3–9. Piscataway, NJ, United States: Innovations in Systems and Software Engineering, IEEE. 10.1109/SMC-IT.2011.14

[btae469-B47] The Galaxy Community. The Galaxy platform for accessible, reproducible and collaborative biomedical analyses: 2022 update. Nucleic Acids Res 2022;50:W345–51. 10.1093/nar/gkac247 35446428 PMC9252830

[btae469-B48] Thelwall M , Kousha K. Academic software downloads from google code: useful usage indicators? Inform Res 2016;21:709.

[btae469-B49] Thomas D , Hunt A. The Pragmatic Programmer, 20th Anniversary Edition. Addison-Wesley, 2019.

[btae469-B50] Waller LA. Documenting and evaluating data science contributions in academic promotion in departments of statistics and biostatistics. Am Stat 2018;72:11–9. 10.1080/00031305.2017.1375988

[btae469-B51] Wang Y. Cognitive complexity of software and its measurement. In: *2006 5th IEEE International Conference on Cognitive Informatics*, Beijing, China Vol. 1. July 2006, 226–35. Piscataway, NJ, United States: IEEE. 10.1109/COGINF.2006.365701

[btae469-B52] Warner JL , Klemm JD. Informatics tools for cancer research and care: bridging the gap between innovation and implementation. JCO Clin Cancer Inform 2020;4:784–6. 10.1200/CCI.20.00086 32870722 PMC7529501

[btae469-B53] Welker KD. Software maintainability index revisited. CrossTalk—J Defense Softw Eng 2001:18–21.

[btae469-B54] Wratten L , Wilm A, Göke J. Reproducible, scalable, and shareable analysis pipelines with bioinformatics workflow managers. Nat Methods 2021;18:1161–8. 10.1038/s41592-021-01254-9 34556866

[btae469-B55] Zhao Y , Liang R, Chen X et al Evaluation indicators for open-source software: a review. Cybersecur 2021;4:1–24.

