# Supplementary material for: Best practices to evaluate the impact of biomedical research software—metric collection beyond citations
Source: Bioinformatics. 2024 Jul 27;40(8):btae469. doi: 10.1093/bioinformatics/btae469 (PMC11297485; doi:10.1093/bioinformatics/btae469)
Supplement: btae469_Supplementary_Data [file btae469_supplementary_data.pdf]

Supplemental Materials

Evaluation of software impact designed for biomedical research: Are we measuring what's meaningful?

Awan Afiaz, Andrey Ivanov, John Chamberlin, David Hanauer, Candace Savonen, Mary J Goldman, Martin Morgan, Michael Reich, Alexander Getka, Aaron Holmes, Sarthak Pati, Dan Knight, Paul C. Boutros, Spyridon Bakas, J. Gregory Caporaso, Guilherme Del Fiol, Harry Hochheiser, Brian Haas, Patrick D. Schloss, James A. Eddy, Jake Albrecht, Andrey Fedorov, Levi Waldron, Ava M. Hoffman, Richard L. Bradshaw, Jeffrey T. Leek, Carrie Wright*

*Correspondence to cwright2@fredhutch.org

Contents

1. **Supplemental Table S1**
2. **Supplemental Notes S1-S5.**

Supplemental Tables

Table S1: **Survey Responses.** Responses to the question, "What would be your goals in evaluating the impact, engagement, or usage of a software tool?" Note that similar responses were considered under the same response category and individual pieces of responses were split to group similar elements of responses together.

**Answer**	**Frequency**
Understand the extent to which a software tool is being used	11
Determining user needs and maximizing utility and impact for target users	8
Accrue information, devise metrics, and measure milestones to support grant reporting and funding	8
Evaluate if the software tool satisfies user requirements	7
Improve software and optimize development	7
Understand ease of use for new users, improve usability	5
Inform efforts, direction of research	4
Identifying popular features	3
Evaluating adaptability and integration with other tools	3
Improve community reach and engagement	2
Understand clinical significance, adoption by healthcare professionals, and effect on patient care	2
"Quantitative assessment and goal setting" and help decide where to focus resources	2
"Understand the value brought by the software and data."	1
"Identify ways to engage a broader community and increase userbase."	1
"Gather data to drive decisions around software practices."	1
"Measuring the usage of individual system components and understanding and planning for periodic fluctuations in demand."	1
Source of motivation to continue building the tool and improving the tool	1
Gather information to support publications	1

Supplemental Notes

S1 Methods

Greater detail, as well as information about data access, and code access about the survey and the manual tool characteristics evaluation can be found at this website.

S1.1 Survey

Forty eight participants (those involved with software development, maintenance, or outreach) in the ITCR program were asked a series of questions about their attitudes on evaluating the impact of scientific software. Some questions were more general, while others asked about practices for their most mature tool. These responses were collected anonymously, however respondents could include a link to their tool if they felt comfortable. The raw survey responses will not be made available to the public to protect the respondents. The survey questions can be found at this webpage.

S1.2 Manual Tool Characteristic Evaluation

Documentation depth: The binary variable "Documentation depth" characterizes the degree of documentation for each tool as either "extensive" or "Webpage/PDF/ReadMe". The term extensive documentation refers to comprehensive resources such as user guides, training, tutorials, code examples, and FAQs that allow users to better understand the functionality of the tools and interpret their outputs. The "Webpage/PDF/ReadMe" category, on the other hand, designates that the tool offers less information, possibly in the form of a website, PDF, or readme file. This "Webpage/PDF/ReadMe" category can be interpreted as having a lower level of documentation depth, as it typically provides users with a more basic level of information about the tool. This type of documentation may only include a brief description of the tool, its features, and how to install it without going into more detailed instructions or use cases. The "Webpage/PDF/ReadMe" category may be sufficient for users who are already familiar with similar tools or have prior experience in the field, but it may not provide enough guidance for beginners or users who need more detailed instructions to use the tool effectively. Therefore,

having a higher level of documentation depth, such as "extensive,", can be beneficial for both novice and experienced users, as it provides more detailed information, tutorials, and use cases, making it easier for them to learn about the tool and utilize it to its full potential.

Instruction on How to cite: The binary variable "Instruction on How to cite" specifies whether each tool provides instructions on how to properly cite it in a research publication or other work. These instructions typically include information such as the author(s) names, version number, publication date, and website or repository where the tool can be found. Tools that do not provide such instructions can make it difficult for researchers to correctly cite the tool, potentially leading to incomplete or inaccurate acknowledgements of its use in research.

Twitter Presence: The variable "Twitter Presence" indicates whether a tool has a formal presence on Twitter. Only 3 tools had other forms of social media including YouTube (two tools - one with twitter and one without) and gitter (one tool with twitter). Tools with a twitter presence have an active account that distributes news, updates, and information on the development, features, and usage of the tool. These accounts can allow communication between tool creators and users while facilitating the tool's exposure and accessibility.

Extensive Contact: The binary variable "Extensive Contact Ways" indicates whether a tool offers multiple ways for users to contact developers for support or other inquiries, such as email addresses, discussion boards, contact forms, and more. Tools with several contact options may be more user-friendly as they enable researchers to seek support or report issues with the tool more conveniently and effectively. In contrast, tools with inadequate or no contact methods may be less attractive to researchers, as they don't provide timely help for technical difficulties or other concerns.

S1.3 Classification of software mention types

Software mentions in scholarly articles can be categorized into four types [1, 2]: allusion, usage, creation, and deposition. The distinction between these types can help researchers understand the role and availability of software in scholarly publications. We distinguish the aforementioned classifications of mention types as follows:

Allusion: This type of mention simply refers to the name of the software and does not require any indication of its usage. Allusions are used to state a

fact about the software or to compare multiple software options for a problem. These are similar to the typical scholarly citations used to refer to related work.

Usage: A usage type mention occurs when software is used in an investigation and contributes to the study. This type of mention allows for conclusions about the study's origins and can be used to develop impact metrics.

Creation: When new software is introduced in a publication, it is a creation type mention. This type of mention can be used to provide scholarly credit to the software's authors, as well as map new creations and track down original software publications.

Deposition: The publication of new software is regarded as a deposition mention. By including information about the publication, such as a license or URL, deposition expands on the creation type mention. When describing availability and licensing information for software, cross-references are used to annotate indirect statements about the software.

In summary, allusion-type mentions provide a broad overview of the presence of software in the text, while usage mentions allow for conclusions regarding its actual contribution. Creation mentions are used to identify newly created software, and deposition mentions provide details on its publication and availability.

Our analyses fully excluded the creation type mentions. We focused on the usage type mentions as this metric facilitates the evaluation of impact of the software tools better than the other mention types.

S1.4 Software Mention Analysis

SoftwareKG-PMC is a database of text-mined software mentions published by [3]. In principle, this resource enables direct identification and analysis of the usage of ITCR tools in published literature present on PubMedCentral Open Access Subset as of January 2021. Access to this data requires setting up the database locally, as described by the developers at this GitHub repository. We downloaded the raw ntriple data from this SoftwareKG-PMC and loaded these into a Virtuoso database using the tenforce/virtuoso Docker image available at this link. We adjusted the virtuoso configuration file for a machine with 64Gb of RAM. After the database is instantiated, the Docker container can be closed and resumed on demand.

We queried the database instance from R using code adapted from the

SofwareKG-PMC-Analysis code notebook. The SPARQL library version 1.16 was installed manually from the CRAN archive at this link as the package is no longer maintained. We constructed a query function which returns all articles which mention a given keyword (i.e., software name) in a particular mention category (allusion, usage, creation, or deposition). Usage was used for most further analyses. This yielded 73095 article-level mentions of any type, corresponding to 60970 unique articles for 36 ITCR tool name keywords. 8 tools were not identified, possibly due to recency of release, complexity of the tool name, or gaps in the accuracy or completeness of SoftwareKG and/or PubMedCentral. We further note that results are contaminated to a varying degree by software name homonyms. This type of collision is a chief limitation of SoftwareKG-PMC, especially for tools with simple or generic names. The query was performed in a case-insensitive manner due to the tendency for authors to adjust the capitalization of software names, such as JBrowse vs Jbrowse. SoftwareKG-PMC does not reliably aggregate these variations.

S2 Survey Qualitative Responses

Supplementary Table S1 shows the responses of several ITCR developers in response to a question about their goals in evaluating their scientific software. The survey participants expressed diverse perspectives, highlighting the wide range of applications for such analyses. The qualitative feedback to the open-ended question was compiled and organized into a tabular format for enhanced readability, providing a comprehensive overview of the developers' inputs about their goals and objectives. Note that direct quotes from the respondents are included with quotation marks.

S3 Software Infrastructure to Enable Evaluation

S3.1 Web presence

Tools vary in terms of web presence. For example, some tools are web-based, some tools simply rely on a README file in a code repository, with simple installation information, while others provide extensive information

and documentation on a separate website. Google Analytics can allow for more fine-grained tracking of how users are interacting with web-based tools or websites and can be helpful for later assessments, in addition to improving access, as one can also perform search engine optimization (SEO) to further encourage the visibility. For example, a developer could track what parts of the documentation users appear to read more frequently and pair this with information about common errors. For web-based tools, great detail can be determined about user interaction. Again Google Analytics can be helpful or for tools using a web-server manual log file inspection, or a service like Cronitor [4] if the tool relies on a web-based server using cron [5] job scheduling can also be informative. It is however important to be mindful of user privacy regulations, see section 4.6.

S3.2 Citability

Another component that can assist with evaluations is providing users with a method of citation, as well as information about how to cite the software, as some users may not be familiar with such practices. This is often done by publishing a manuscript about the software in a scientific journal and providing information about the publication on the software website and code repository. In addition, digital object identifiers (DOI), from publishing platforms like Zenodo [6], can be used for other less conventionally cited materials, such as documentation, case studies, data, and the actual software itself.

Services like Altmetric [7], allow for deeper analysis of engagement for anything with a DOI. They provide reports and badges that can be added to websites or manuscripts (depending on where they are published) that indicate how often a DOI is cited in multiple sources besides scientific articles, such as blogs, news articles, Wikipedia, social media, and more. Individuals or organizations can use Altmetric to get such reports by simply searching for the DOI. These reports also have links to individual social media posts, citing articles, and more. In addition, Semantic Scholar [8] provides reports that indicate where citations have occurred within scientific articles.

S3.3 Documentation

Documenting software not only helps guide users but can also enable collection of useful metrics. Documentation on websites can be tracked and provide

detailed information about such usage. If a particular page has more traffic, it may indicate that the aspect of the tool discussed on that page is either particularly popular or particularly confusing. As with all metrics, pairing information together can aid interpretation. Providing different types of documentation is another step that assures confidence in software, and enables varying roles to succeed in using the software. For example, command-line usage is important for the users of software, while API documentation is important for developers who may want to extend the software.

S3.4 Communications

Providing mechanisms for users to communicate with one another and with the developers can provide another avenue for understanding usage and engagement.

S3.4.1 Feedback mechanisms

One very helpful method of obtaining software usage metrics is to have users directly provide feedback. Providing such feedback mechanisms helps to identify software weaknesses. However, few users provide feedback and often feedback requires interpretation. Individuals are also unlikely to provide positive feedback. Nevertheless, user feedback mechanisms can be powerful for gaining insight from users. Feedback mechanisms can be passive or active in nature. More passive mechanisms may involve providing an email address on a website or simply allowing users to post an issue on a GitHub repository. More active mechanisms may include usability testing interviews, Google forms or other surveys, or providing automated GitHub issue templates to encourage specific kinds of feedback.

S3.4.2 Email and support forums

While it's possible to collect user feedback and provide support through email, if a public support forum is used instead, users can learn from answers to questions of other users, reducing the burden on developers, and provide another opportunity for easier tracking of software engagement. This also provides an opportunity to build a community around the software and to learn what is and isn't working for users. Some forum platforms support upvoting (where other forum users can upvote questions or feature requests). Using an

existing support forum platform such as Discourse (`https://www.discourse.org/`) provides many features that are convenient in forum moderation, such as user trust levels, categorization and tagging of topics, and detailed tracking of forum posts, forum post views, and user and moderator activity levels that serve as useful metrics about a software project and the community around it. This has been especially useful for the QIIME 2 [9] developers.

Engaging with users about what new features they are excited about and acting on those discussions, is a great way to get informed feedback while rewarding user involvement. To further support community, developers could consider inviting user to attend workshops on new features or inviting them to help teach workshops on fundamentals. Forum members who provide intellectual contributions to projects, including technical support, feature suggestions, highly referenced posts, etc, should be included as authors on relevant papers. And public facing activity summaries (e.g., `https://forum.qiime2.org/u/gregcaporaso/summary`) can also be useful for resume building for early career contributors.

Emailing newsletters to registered users, can be a useful method for informing users about new features, updates or issues. Systems like Mailchimp [10] or HubSpot [11] can allow analytics about how often recipients open the news letter, click on links, or unsubscribe.

S3.5 Usability testing

Usability refers to the ease of use for software. Usability testing is a method of purposefully investigating the usability of a software tool. Usability testing can be powerful for collecting valuable metrics and information regarding usage. It involves applying the scientific method to investigating a user's experience with a software tool while the tester observes their interactions with the tool.

Often research institutions do not have experts in user design and testing on staff. However, developers can and should utilize these techniques to conduct their own informal usability testing. Step-by-step guides of how to conduct usability testing have been published elsewhere [12]. It should be noted that the benefits of usability testing are high even when only a few users are observed.

S3.6 Workshops

Hands-on workshops (online[13] or in-person) for your software are an excellent way to build a user community and get feedback. Workshops allow developers to identify what confuses or challenges users. The challenges are often not what developers expect. Attendees can also be asked to participate in surveys about what they like and don't like to help guide future development. The quantity, duration, and attendance at your workshops are metrics that can be reported to funding agencies in grant proposals or reports. Posting recordings of events can be shared on YouTube or other platforms, which allows for other useful metrics.

S3.7 Code of conduct

Before engaging with user or developer communities, it is important to develop and publish a code of conduct to outline expectations of behavior and how contributors can report violations. A code of conduct can be reassuring of community health and are required by some scientific software funders, such as the Chan Zuckerberg Initative's Essential Open Source Software for Science program. Thus the presence of a Code of Conduct may be a measure that is assessed by potential funders. A good starting point for a code of conduct is the Contributor Covenant (https://www.contributor-covenant.org/), which can be adapted. Adopting and effectively managing[14] a Code of Conduct can support the growth of your community by indicating a safe space, thereby encouraging engagement by individuals who might feel intimidated about getting publicly involved.

S3.8 Social media

Having a social media presence through platforms such as twitter, instagram, and youtube, provides opportunities to track engagement with social media posts. Pairing this with evaluations of engagement with the software itself, to determine if outreach strategies are successful. This can also be helpful to determine if documentation resources are useful. For example, the number of video views on a youtube documentation video can be informative to know what percentage of users may have actually seen the documentation. Videos with many views can also reassure others about using the software .

S3.9 Reviews

There are review mechanisms that can help reassure users about software. For example, SourceForge[15] a platform for publishing and developing software allows users to rate software on the platform. Developers can integrate their GitHub repositories with SorceForge to take advantage of this review platform. Alternatively, GitHub also has a system of adding stars to or "watching" repositories [16], however, this appears to be somewhat inconsistently done in the community.

S3.10 Version control mechanisms

Many bioinformatics software projects maintain their source code under public revision or version control, using a platform such as GitHub or GitLab. Provided that the platform is actually used for version control (as opposed to hosting one or two versions of the software), this is a useful indicator of project health. Users can be confident that changes to the code are tracked to some extent and can also observe how much activity and updates have occurred to the software – this can indicate to the user and developers of the project how recently maintenance has occurred. This can also help users identify the specific version or revision of the software they are using to record and report their methods, reproduce an analysis, or determine if they are impacted by a bug in the software. Furthermore, GitHub provides an insight tab with information about development metrics and its API allows for collection of these metrics in a systematic and reproducible way.

S4 Software Health Infrastructure

S4.1 Development activity

Finally project health can be assessed based on activity. By reviewing the frequency of commits and the date of the most recent commit in a revision control system, it is possible to assess whether the project is actively developed. Inactively developed projects project may not include fixes for recently discovered bugs, or improvements in methods to keep current with the field. Tracking who is making commits to the project can also provide details on whether the project is the work of one or a few developers, or of a large and active developer community. Neither a big or small developer

community is necessarily better, though smaller developer communities can pose greater sustainability risk. If there is only one person who knows how to work on the code, and that person becomes unable or unwilling to continue working on the project, development or maintenance of the project may be discontinued.

S4.2 Automated test coverage and continuous integration testing

Bugs in scientific software can have far-reaching impacts. Automated software tests allow developers to confirm that their code is behaving as expected under a range of normal and abnormal conditions. Systematically developed tests that can be run by both developers and users with a single command, give confidence that the software is doing what it is supposed to be doing. Platforms such as GitHub allow developers to advertise their test code coverage (the fraction of lines of code in the project that are covered by automated tests) and current build status (including whether tests are currently passing or failing). The presence or absence of this information, as well as quantitative metrics like test code coverage, are useful indicators of project health. Similarly, the use of automated or continuous integration testing, which can be configured to run when a change is committed to a software repository, when software releases are created, or at any other point where quality assurance is necessary to provide early signals of errors, is also an indicator of project health. This can help ensure that bugs or other issues can be detected before software is deployed by users.

The presence of different types of automated tests can be seen as an indicator of software maturity, and the care taken in the process of developing the software. Unit tests provide confidence in individual pieces of code; component and integration tests provide confidence that individual pieces of code behave correctly together; acceptance tests provide confidence that the overall software behaviors are correct. Achieving this in-depth test coverage requires careful software design upfront and throughout development. It is worth noting that, while useful, test coverage is not always a trustworthy representation of test quality. It is simply a measure of the proportion of code which is run by the test suite. It does not evaluate the quality of the test cases or assertions.

Other related static code analysis metrics can be used in conjunction

with test coverage to build a more well-rounded picture of code quality. For example, cyclomatic complexity measures the logical complexity of a function. Code with high cyclomatic complexity contains numerous branching paths with variations in execution, and therefore should be avoided. A complex function can have 100% test coverage without coming close to testing all possible outcomes. In fact, writing tests for highly complex code is often a lost cause. By considering cyclomatic complexity alongside test coverage, programmers can target specific areas of a codebase that need more tests. However, they can also see where they should instead prioritize refactoring, reducing complexity and improving testability first.

S4.3 Licensing

In general, a software project should have a discoverable license that allows users to understand how they are and are not allowed to use the software. Licenses differ in how permissive they are, but the lack of a license defaults to highly restrictive terms and is an indicator that use of the software by others hasn't been adequately considered. Similarly, the choice of license can be another indicator of inadequate usage consideration due to the restrictive and binding nature of some software licenses.

S5 Security

In any repository that contains source code, it is of vital importance that authentication secrets, such as API keys or cryptographic secrets be managed properly [17]. It becomes even more important in the case of code that deals with healthcare data, where even the slightest mishap in this regard could potentially expose protected health information (PHI) data and/or information to the public. During the continuous integration (CI) step of any project, incorporation of reputed static code analysis tools [18, 19] (which are often available to open-source projects at no cost) ensures that tracking of the perpetually evolving landscape of security threats is offloaded to an entity that is specialized for its detection. It also allows the code to scale at a quick pace while ensuring bugs can be identified early in the development process, and finally allows software architects to define security guidelines to assist developers without impeding on their development process. It is understood and accepted that open-source code is built "on the shoulders of others", and reg-

ular analysis of the underlying dependencies (such as using the "Dependabot" product from GitHub [20]) allows the automatic detection of security vulnerabilities (and potential mitigation strategies presented) to the development team in question [21]. Products such as Dependabot maintain a frequently updated collection of packages with version/commit identifiers that contain security vulnerabilities with their fixes as defined by the broader community, and as such should be incorporated into all open-source repositories. Another set of security considerations come when packaging and deploying code in the form of containers, and more specifically using Dockers [17]. Depending on the veracity of the adversary in question, attacks on Docker containers can range from targeting the container itself, the host system, any collocated containers, the container management system or the source code itself[22]. One of the most common security threats from Docker containers come in the form of elevated user access [22, 23], which can be a critical flaw when running containers in an High Performance Computing (HPC) environment [24, 25]. This is specifically addressed using other containerization protocols such Singularity [26] and Podman [27]. Though a single solution might not work for all cases, when deploying open-source software using the Docker containerization protocol, specific security mitigation strategies should be considered [28].

S6 References

[1] David Schindler, Kristina Yordanova, and Frank Krüger. An annotation scheme for references to research artefacts in scientific publications. In *2019 IEEE International Conference on Pervasive Computing and Communications Workshops (PerCom Workshops)*, pages 52–57, March 2019.

[2] David Schindler, Felix Bensmann, Stefan Dietze, and Frank Krüger. SoMeSci- A 5 Star Open Data Gold Standard Knowledge Graph of Software Mentions in Scientific Articles, August 2021. arXiv:2108.09070 [cs].

[3] Frank Krüger and David Schindler. A Literature Review on Methods for the Extraction of Usage Statements of Software and Data. *Computing in Science & Engineering*, 22(1):26–38, January 2020. Conference Name: Computing in Science & Engineering.

[4] Cronitor. https://cronitor.io/.

[5] Ron Peters. cron. in: Expert shell scripting. In Ron Peters, editor, *Expert Shell Scripting*, pages 81–85. Apress, Berkeley, CA, 2009.

[6] Zenodo - Research. Shared.

[7] Altmetric badges. https://www.altmetric.com/products/altmetric-badges/, July 2015.

[8] Semantic Scholar | AI-Powered Research Tool. https://www.semanticscholar.org/.

[9] Evan Bolyen, Jai Ram Rideout, Matthew R. Dillon, Nicholas A. Bokulich, Christian C. Abnet, Gabriel A. Al-Ghalith, Harriet Alexander, Eric J. Alm, Manimozhiyan Arumugam, Francesco Asnicar, Yang Bai, Jordan E. Bisanz, Kyle Bittinger, Asker Brejnrod, Colin J. Brislawn, C. Titus Brown, Benjamin J. Callahan, Andrés Mauricio Caraballo-Rodríguez, John Chase, Emily K. Cope, Ricardo Da Silva, Christian Diener, Pieter C. Dorrestein, Gavin M. Douglas, Daniel M. Durall, Claire Duvallet, Christian F. Edwardson, Madeleine Ernst, Mehrbod Estaki, Jennifer Fouquier, Julia M. Gauglitz, Sean M. Gibbons, Deanna L. Gibson, Antonio Gonzalez, Kestrel Gorlick, Jiarong Guo, Benjamin Hillmann, Susan Holmes,

Hannes Holste, Curtis Huttenhower, Gavin A. Huttley, Stefan Janssen, Alan K. Jarmusch, Lingjing Jiang, Benjamin D. Kaehler, Kyo Bin Kang, Christopher R. Keefe, Paul Keim, Scott T. Kelley, Dan Knights, Irina Koester, Tomasz Kosciolek, Jorden Kreps, Morgan G. I. Langille, Joslynn Lee, Ruth Ley, Yong-Xin Liu, Erikka Loftfield, Catherine Lozupone, Massoud Maher, Clarisse Marotz, Bryan D. Martin, Daniel McDonald, Lauren J. McIver, Alexey V. Melnik, Jessica L. Metcalf, Sydney C. Morgan, Jamie T. Morton, Ahmad Turan Naimey, Jose A. Navas-Molina, Louis Felix Nothias, Stephanie B. Orchanian, Talima Pearson, Samuel L. Peoples, Daniel Petras, Mary Lai Preuss, Elmar Pruesse, Lasse Buur Rasmussen, Adam Rivers, Michael S. Robeson, Patrick Rosenthal, Nicola Segata, Michael Shaffer, Arron Shiffer, Rashmi Sinha, Se Jin Song, John R. Spear, Austin D. Swafford, Luke R. Thompson, Pedro J. Torres, Pauline Trinh, Anupriya Tripathi, Peter J. Turnbaugh, Sabah Ul-Hasan, Justin J. J. van der Hooft, Fernando Vargas, Yoshiki Vázquez-Baeza, Emily Vogtmann, Max von Hippel, William Walters, Yunhu Wan, Mingxun Wang, Jonathan Warren, Kyle C. Weber, Charles H. D. Williamson, Amy D. Willis, Zhenjiang Zech Xu, Jesse R. Zaneveld, Yilong Zhang, Qiyun Zhu, Rob Knight, and J. Gregory Caporaso. Reproducible, interactive, scalable and extensible microbiome data science using QIIME 2. *Nature Biotechnology*, 37(8):852–857, August 2019. Publisher: Nature Publishing Group.

[10] Marketing smarts for big ideas. https://mailchimp.com/.

[11] HubSpot. Free Email Tracking Software | HubSpot. https://www.hubspot.com/products/sales/email-tracking.

[12] Candace Savonen. Documentation and usability, 2021.

[13] Matthew R. Dillon, Evan Bolyen, Anja Adamov, Aeriel Belk, Emily Borsom, Zachary Burcham, Justine W. Debelius, Heather Deel, Alex Emmons, Mehrbod Estaki, Chloe Herman, Christopher R. Keefe, Jamie T. Morton, Renato R. M. Oliveira, Andrew Sanchez, Anthony Simard, Yoshiki Vázquez-Baeza, Michal Ziemski, Hazuki E. Miwa, Terry A. Kerere, Carline Coote, Richard Bonneau, Rob Knight, Guilherme Oliveira, Piraveen Gopalasingam, Benjamin D. Kaehler, Emily K. Cope, Jessica L. Metcalf, Michael S. Robeson Ii, Nicholas A. Bokulich, and J. Gregory Caporaso. Experiences and lessons learned from two virtual,

hands-on microbiome bioinformatics workshops. *PLoS computational biology*, 17(6):e1009056, June 2021.

[14] Valerie Aurora and Mary Gardiner. *How to Respond to Code of Conduct Reports*. Frame Shift Consulting LLC https://frameshiftconsulting.com, 1.1 edition, 2019.

[15] sourceforge: The complete open-source and business software platform. https://sourceforge.net/.

[16] Mikhail G. Dozmorov. GitHub Statistics as a Measure of the Impact of Open-Source Bioinformatics Software. *Frontiers in Bioengineering and Biotechnology*, 6, 2018.

[17] Dirk Merkel. Docker: Lightweight Linux Containers for Consistent Development and Deployment.

[18] P. Louridas. Static code analysis. *IEEE Software*, 23(4):58–61, July 2006. Conference Name: IEEE Software.

[19] Jeremy Ludwig, Steven Xu, and Frederick Webber. Compiling static software metrics for reliability and maintainability from GitHub repositories. In *2017 IEEE International Conference on Systems, Man, and Cybernetics (SMC)*, pages 5–9, October 2017.

[20] Dependabot. https://github.com/dependabot.

[21] Mahmoud Alfadel, Diego Elias Costa, Emad Shihab, and Mouafak Mkhallalati. On the Use of Dependabot Security Pull Requests. In *2021 IEEE/ACM 18th International Conference on Mining Software Repositories (MSR)*, pages 254–265, May 2021. ISSN: 2574-3864.

[22] Theo Combe, Antony Martin, and Roberto Di Pietro. To Docker or Not to Docker: A Security Perspective. *IEEE Cloud Computing*, 3(5):54–62, September 2016. Conference Name: IEEE Cloud Computing.

[23] Thanh Bui. Analysis of Docker Security, January 2015. arXiv:1501.02967 [cs].

[24] Jonathan Sparks. Enabling Docker for HPC. *Concurrency and Computation: Practice and Experience*, 31(16):e5018, 2019. _eprint: https://onlinelibrary.wiley.com/doi/pdf/10.1002/cpe.5018.

[25] Enrico Bacis, Simone Mutti, Steven Capelli, and Stefano Paraboschi. DockerPolicyModules: Mandatory Access Control for Docker containers. In *2015 IEEE Conference on Communications and Network Security (CNS)*, pages 749–750, September 2015.

[26] Gregory M. Kurtzer, Vanessa Sochat, and Michael W. Bauer. Singularity: Scientific containers for mobility of compute. *PLOS ONE*, 12(5):e0177459, May 2017.

[27] Holger Gantikow, Steffen Walter, and Christoph Reich. Rootless Containers with Podman for HPC. In Heike Jagode, Hartwig Anzt, Guido Juckeland, and Hatem Ltaief, editors, *High Performance Computing*, Lecture Notes in Computer Science, pages 343–354, Cham, 2020. Springer International Publishing.

[28] Robail Yasrab. Mitigating Docker Security Issues, August 2021. arXiv:1804.05039 [cs].
